# Investigation of interfractional range variation owing to anatomical changes with beam directions based on water equivalent thickness in proton therapy for pancreatic cancer

**DOI:** 10.1093/jrr/rrae069

**Published:** 2024-10-08

**Authors:** Yuhei Kikkawa, Hideaki Ueda, Yusuke Uchinami, Norio Katoh, Hidefumi Aoyama, Yoichi M Ito, Kohei Yokokawa, Ye Chen, Taeko Matsuura, Naoki Miyamoto, Seishin Takao

**Affiliations:** Graduate School of Engineering, Hokkaido University, North13 West8, Kita-ku, Sapporo, Hokkaido 0608628, Japan; Faculty of Engineering, Hokkaido University, North13 West8, Kita-ku, Sapporo, Hokkaido 0608628, Japan; Faculty of Medicine, Hokkaido University, North15 West7, Kita-ku, Sapporo, Hokkaido 0608638, Japan; Faculty of Medicine, Hokkaido University, North15 West7, Kita-ku, Sapporo, Hokkaido 0608638, Japan; Faculty of Medicine, Hokkaido University, North15 West7, Kita-ku, Sapporo, Hokkaido 0608638, Japan; Institute of Health Science Innovation for Medical Care, Hokkaido University Hospital, North14 West5, Kita-ku, Sapporo, Hokkaido 0608648, Japan; Department of Medical Physics, Hokkaido University Hospital, North14 West5, Kita-ku, Sapporo, Hokkaido 0608648, Japan; Faculty of Engineering, Hokkaido University, North13 West8, Kita-ku, Sapporo, Hokkaido 0608628, Japan; Faculty of Engineering, Hokkaido University, North13 West8, Kita-ku, Sapporo, Hokkaido 0608628, Japan; Faculty of Engineering, Hokkaido University, North13 West8, Kita-ku, Sapporo, Hokkaido 0608628, Japan; Faculty of Engineering, Hokkaido University, North13 West8, Kita-ku, Sapporo, Hokkaido 0608628, Japan

**Keywords:** interfractional range variation, water equivalent thickness, pancreatic cancer, proton therapy

## Abstract

To assess the interfractional anatomical range variations (ARVs) with beam directions and their impact on dose distribution in intensity modulated proton therapy, we analyzed water equivalent thickness (WET) from 10 patients with pancreatic cancer. The distributions of the interfractional WET difference ($\Delta{\mathrm{WET}}^{\theta }$) across 360° were visualized using polar histograms. Interfractional ARVs were evaluated using the mean absolute error and ΔWET pass rate, indicating the percentage of $\Delta \mathrm{WE}{\mathrm{T}}^{\theta }$ < thresholds. The impact on dose distribution in proton therapy was evaluated based on two treatment plans for 40 Gy(RBE)/5 fractions: ‘Plan A’, using two beam angles, in which the target was closest to the body surface among four perpendicular directions; and ‘Plan B’, using two beam angles with small ARVs. Analysis revealed individual variations in angular trends of interfractional ARVs. Three distinct trends were identified: Group 1 exhibited small ARVs around posterior directions; Group 2 exhibited small ARVs except ~60°; Group 3 demonstrated minimal ARVs only ~90°. In dose evaluation, while 150° and 210° were selected in Plan B for 9 out of 10 patients, for the remaining patient, 60° and 90° were chosen. Comparing dose volume histogram parameters for all patients, Plan B significantly reduced target coverage loss while maintaining organ-at-risk sparing comparable to Plan A. These results demonstrated that selecting beam angles with small interfractional ARVs for each patient enhances the robustness of dose distribution, reducing target coverage loss.

## INTRODUCTION

Pancreatic cancer has one of the lowest 5-year survival rates of <10% [1]. Surgical resection is the primary treatment option for resectable cases but only accounts for ~20% of all pancreatic cancer cases; therefore, radiation therapy is important for unresectable cases, such as locally advanced pancreatic cancer [2]. In radiation therapy, sufficient doses must be delivered to the target while sparing the organs-at-risk (OARs). The dose distribution of charged particles, such as protons and carbon ions, is characterized by a sharp distal fall-off, allowing a more conformal dose distribution compared with that of photon therapy. In charged particle therapy, a conformal dose distribution can be beneficial for treatment of pancreatic cancer surrounded by OARs, such as the gastrointestinal (GI) tract. Improvement in local control rates in proton therapy for pancreatic cancer has been reported while maintaining acceptable toxicities to OARs [3]. However, the dose distribution in proton therapy is highly sensitive to range uncertainties, and a beam-specific margin or robust optimization in proton therapy planning can help to mitigate range uncertainties. When adding a beam-specific margin, the treatment volume is generally extended in the beam direction to account for range calculation errors [4]. During robust optimization, the irradiation dose is optimized to account for setup errors and range calculation errors [5, 6]. However, accounting for the interfractional range variations caused by anatomical changes throughout treatment course is difficult; these variations must be maintained below an acceptable level to fully realize the potential of proton therapy [7, 8].

The interfractional range variation based on the water equivalent thickness (WET) has been investigated in lung, prostate, and head and neck cancer cases [9, 10]. The interfractional WET difference (ΔWET) exhibits a significant correlation with target coverage in intensity modulated proton therapy (IMPT) for patients with lung cancer, where the impact of the interfractional range variation on proton therapy was evaluated at typical beam angles [11]. However, the range variations in all 360° directions must be investigated to determine the most appropriate beam direction. Jihun *et al.* investigated angular range variation in patients with head and neck cancer and concluded that the range variation for the posterior oblique beam was sensitive to patient weight loss and setup errors [12]. Yuan *et al.* proposed beam configurations that minimize the mean absolute error (MAE) of angular WET in carbon-ion radiation therapy for pancreatic cancer, which was found to effectively enhance target coverage [13]. Nonetheless, incorporating individual interfractional range variations into the robust selection of beam directions for each patient still remains challenging, primarily because pancreatic tumors are surrounded by the GI tract, which considerably varies from day-to-day and patient-to-patient in terms of shape and content.

Herein, we defined anatomical range variations (ARVs) as the range variations caused by anatomical changes. We investigated the interfractional ARVs with beam directions based on ΔWET in proton therapy for pancreatic cancer. We also evaluated the impact of interfractional ARVs on proton dose distribution with different beam directions selected based on the ARV investigations. Our study provides further information on beam angle selection and robust proton therapy through individual assessment of interfractional ARVs with beam directions.

## MATERIALS AND METHODS

### Patient characteristics

This study was approved by the Ethics Review Committee of Hokkaido University Hospital (IRB number: 019-0397). We analyzed interfractional ARVs using data from 10 patients with pancreatic cancer who had received stereotactic body radiotherapy (SBRT) of 40 Gy/ 5 fractions at our institution between 1 September 2020 and 31 July 2022. Each patient had a set of planning CT (pCT) images and five sets of daily CT (dCT) images. All dCT images were acquired using the same CT scanner as the pCT images. Each patient had 6 hours or more of fasting time before pCT and dCT scans. All pCT and dCT images were acquired with a voxel spacing of 0.977 × 0.977 × 2.0 mm^3^ and axial plane image sizes of 512 × 512 (500 × 500 mm^2^) during end-exhaled breath holding. A gold fiducial marker was implanted near the tumor in each patient. The patient characteristics are summarized in Table 1.

**Table 1:** Summary of patient characteristics

				Target volume [cc]		Elapsed days after pCT [day]				
Patient	Age	Sex	Target location	GTV	CTV	Fx 1	Fx 2	Fx 3	Fx 4	Fx 5
1	89	F	Body	4.8	8.2	13	15	19	20	22
2	73	M	Body	2.5	3.5	8	11	12	14	18
3	79	M	Body	14.8	16.8	11	13	14	18	19
4	81	M	Head	9.0	12.7	11	13	15	18	19
5	50	M	Tail	4.0	5.6	12	14	16	19	20
6*	78	M	Head	1.2	2.2	12	14	18	19	21
7	71	M	Head	2.6	4.5	9	10	14	15	17
8*	75	M	Head	16.7	20.7	17	19	21	24	25
9	84	F	Head	4.3	5.9	13	14	16	19	20
10	88	M	Body	7.8	11.9	12	13	15	18	19

Abbreviations: *, patient who had received surgery; M, male; F, female; Body, pancreatic body; Head, pancreatic head; Tail, pancreatic tail; Fx, fraction.

### Image preprocessing

During proton therapy, patient positioning process involved bone matching, followed by fiducial marker matching [14]. Daily CT images were aligned with pCT according to this positioning process using in-house software. First, dCT images were moved in six degrees of freedom (translation and rotation) to maximize the normalized mutual information within the rectangular area containing the spine [15]. Subsequently, these bone-matched dCT images were translationally adjusted to align the marker position on these images with that on the pCT images.

### Calculation of WET differences

The daily range errors were evaluated based on ΔWET at beam angle $\theta$ ($\Delta \mathrm{WE}{\mathrm{T}}^{\theta }$), which was calculated from pCT and dCT images, according to the following equation [16]:


$$ \Delta{\mathrm{WET}}^{\theta }\!=\!\!{\int}_0^{d_{\mathrm{iso}}}\mathrm{SPR}\left({\mathrm{HU}}_{\mathrm{dCT}}\left({z}^{\theta}\right)\right)d{z}^{\theta }-\!{\int}_0^{d_{\mathrm{iso}}}\mathrm{SPR}\left({\mathrm{HU}}_{\mathrm{pCT}}\left({z}^{\theta}\right)\right)d{z}^{\theta } $$

where ${d}_{iso}$ is the depth of the isocenter, ${\mathrm{HU}}_{\mathrm{pCT}}(z)$ and ${\mathrm{HU}}_{\mathrm{dCT}}(z)$ are the Hounsfield units of the voxel at depth ${z}^{\theta }$ on pCT and dCT images. $\mathrm{SPR}\left(\mathrm{HU}\right)$ is the stopping power ratio converted from the Hounsfield unit. The daily $\Delta \mathrm{WE}{\mathrm{T}}^{\theta }$was analyzed over all beam angles, as described in the next section.

### Evaluation of interfractional ARVs based on WET differences

**Fig. 1:**
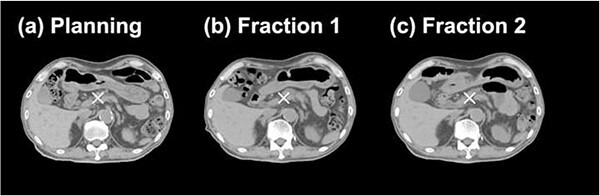
Axial slices including isocenter of (**a**) pCT images, and dCT images at (**b**) fraction 1 and (**c**) fraction 2 for Patient 2. The dCT images were aligned by the fiducial marker position. The white crosses indicate the isocenter on each CT image.

**Fig. 2:**
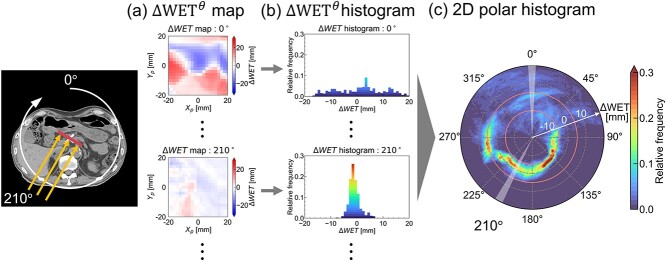
An overview of the analysis schematic. (**a**) ΔWET was calculated up to isoplane in the evaluation region of $\pm$20 mm centered at the isocenter to obtain ΔWET$^{\boldsymbol{\theta} }$ distribution. (**b**) ΔWET$ {}^{\boldsymbol{\theta}}$ histogram was created from ΔWET$ {}^{\boldsymbol{\theta}}$ distribution. (**c**) We repeated the same procedure for all beam directions and summarized in a polar coordinate 2D histogram. Two orange lines in the 2D polar histograms represent ΔWET$ {}^{\boldsymbol{\theta}}$ = $\pm 5$ mm.

Figure 1 shows the CT images for Patient 2 after the marker-based alignment. Anatomical changes were clearly observed, especially in the GI tract. Figure 2 illustrates the individual ΔWET analysis schematic. The daily range variations were evaluated based on the distribution of $\Delta \mathrm{WE}{\mathrm{T}}^{\theta }$ calculated in a 40 × 40-mm^2^ evaluation area on the isoplane, which was determined to be sufficient to include the clinical target volume (CTV) for the 10 patients. Since $\Delta \mathrm{WE}{\mathrm{T}}^{\theta }$ was calculated at 2-mm intervals, a $\Delta \mathrm{WE}{\mathrm{T}}^{\theta }$ distribution represents an array of 441 calculation points (21 × 21). A total of 360 $\Delta \mathrm{WE}{\mathrm{T}}^{\theta }$ distributions were generated as $\Delta \mathrm{WE}{\mathrm{T}}^{\theta }$ map at 1° intervals (Fig. 2a ). Subsequently, the $\Delta \mathrm{WE}{\mathrm{T}}^{\theta }$ histograms obtained from the $\Delta \mathrm{WE}{\mathrm{T}}^{\theta }$ maps (Fig. 2b ) were summarized into a 2D polar histogram (Fig. 2c ). The angular axis and the radial axis represent the beam angle $\theta$ (deg) and $\Delta \mathrm{WE}{\mathrm{T}}^{\theta }$ (mm), respectively.

The MAE of the ${\mathrm{WET}}^{\theta }$ (${\mathrm{MAE}}_{\mathrm{WET}}^{\theta }$) and ΔWET pass rate (${\mathrm{R}}_{\mathrm{pass}}^{\theta }$), representing the percentage of $\Delta \mathrm{WE}{\mathrm{T}}^{\theta }$ < thresholds, were calculated per beam angle to quantitatively evaluate interfractional ARVs:


$$ {\mathrm{MAE}}_{\mathrm{WET}}^{\theta }=\frac{1}{N_{\mathrm{cp}}}{\sum}_j^{N_{\mathrm{cp}}}\left|\Delta{\mathrm{WET}}_j^{\theta}\right| $$

$$ {\mathrm{R}}_{\mathrm{pass}}^{\theta }=\frac{N_{\mathrm{pass}}^{\theta }}{N_{\mathrm{cp}}}\times 100 $$

where $ \theta$ is the beam directions from 0° to 360°,$\kern0.5em {N}_{\mathrm{cp}}=441$ is the number of calculation points on the $\Delta \mathrm{WE}{\mathrm{T}}^{\theta }$ map, and ${N}_{\mathrm{pass}}$ is the number of points where the $\Delta \mathrm{WE}{\mathrm{T}}^{\theta }$ is less than the thresholds (5, 7 or 10 mm). To evaluate the interfractional ARVs at typical beam angles ${\theta}_{\mathrm{typ}}\in \left\{{0}^{{}^{\circ}},{30}^{{}^{\circ}},{60}^{{}^{\circ}},\dots, {330}^{{}^{\circ}}\right\}$ for proton therapy, the angular average ${\mathrm{MAE}}_{\mathrm{WET}}^{\theta }$ (${\overline{\mathrm{MAE}}}_{\mathrm{WET}}^{\theta_{typ}}$) was calculated as the mean value of the ${\mathrm{MAE}}_{\mathrm{WET}}^{\theta }$ within ±15° at ${\theta}_{\mathrm{typ}}$. Similarly, the mean of angular average ${\mathrm{R}}_{pass}^{\theta }$ (${\overline{\mathrm{R}}}_{pass}^{\theta_{typ}}$) was calculated to assess the interfractional ARVs at ${\theta}_{\mathrm{typ}}$.

### Proton treatment planning and daily dose evaluation

Herein, we evaluated the impact of interfractional ARVs on dose distribution in IMPT for 10 patients. The commercial treatment planning system VQA (Hitachi, Ltd, Tokyo, Japan) was used for planning. We fixed the relative biological effectiveness (RBE) at 1.1. The regions of interest (ROIs) were contoured by experienced radiation oncologists. A dose of 40 Gy(RBE) was prescribed in five fractions with two-field IMPT. For each patient, we created two plans: ‘Plan A’, using two beam angles, in which the target was closest to the body surface among four perpendicular directions to minimize the volume of normal tissue the beam passes through; and ‘Plan B’, using beam angles with small interfractional ARVs from the ${\theta}_{\mathrm{typ}}$. The worst-case robust optimization was used to account for a setup error of ±5 mm in each orthogonal direction and a range uncertainty of ±3.5%.

Forward dose calculation was performed on the dCT images aligned with pCT images by the fiducial marker position. Assuming that the prescribed dose was delivered in each fraction, this enabled us to evaluate the impact of anatomical changes on dose distribution in five different anatomical situations per patient. Subsequently, the daily differences of dose-volume histogram (DVH) parameters between Plans A and B were compared to assess the impact of different ARVs on the dose distribution. The dose constraints were as follows: ${D}_{50\%}$  $\ge$38 Gy(RBE) for gross tumor volume (GTV), ${D}_{99\%}$  $\ge$30 Gy(RBE) for the CTV, ${V}_{33\mathrm{Gy}}$  $<$0.5 cc for the OARs (stomach, duodenum, small bowel, large bowel) and ${V}_{38\mathrm{Gy}}$  $<$0.5 cc for the expanded OARs region with an additional 5-mm margin (OARs +5 mm) [17]. We performed a paired t-test to statistically analyze the differences in mean value of DVH parameters for the 5 days between Plans A and B.

## RESULTS

### Interfractional ARVs with beam directions

**Fig. 3:**
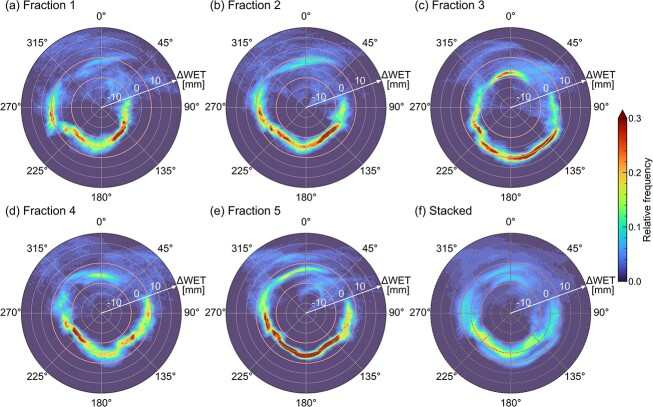
The 2D polar histograms for Patient 2. The graphs of (**a**)–(**e**) represent results from five dCT images. The graph of (**f**) shows the relative frequency of the stacked histogram for all days. The interfractional range variations are obviously small at posterior beam directions.

**Fig. 4:**
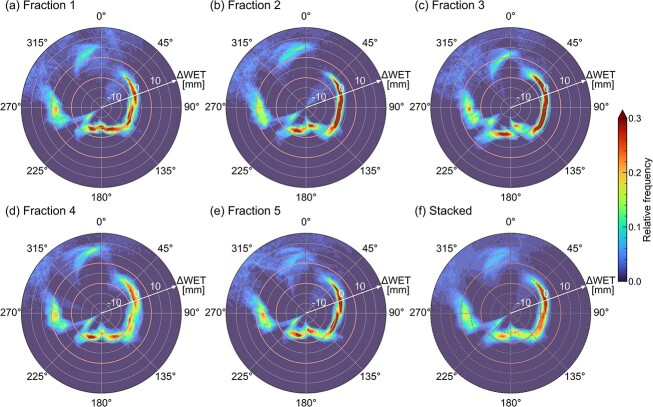
The 2D polar histograms for Patient 5 who had the target in pancreatic tail. Similar to Fig. 3, panels (a)–(e) show the daily results, and panel (f) represents the stacked histogram. The interfractional ARVs were small $\boldsymbol{\sim\!{90}{{}^{\circ}}}$, because of the shallow location of the target viewed from the left lateral directions.

**Fig. 5:**
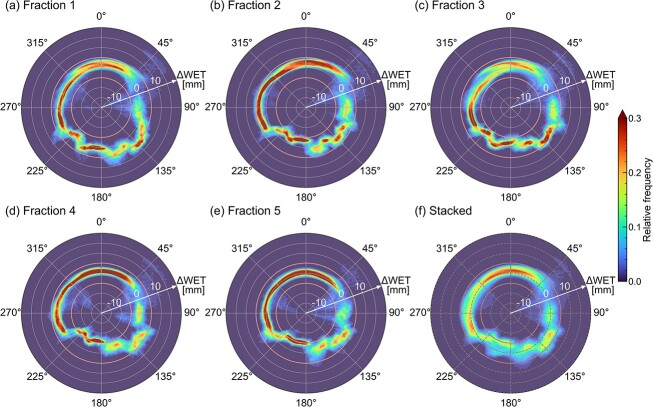
The 2D polar histograms for Patient 8. As in Figs. 3, 4, panels (a)–(e) show the daily results and panel (f) represents the stacked histogram. The GI tract volume on the beam path around the anterior direction was small due to the surgical resection of gastric cancer, and the interfractional ARVs were small at the right lateral and anterior beam angles other than the posterior.

The 2D polar histograms for three representative patients are shown in Figs 3–5. The daily 2D polar histograms (Figs 3–5a–e) show the distribution of daily $\Delta \mathrm{WE}{\mathrm{T}}^{\theta }$ in the evaluation area at each beam direction. Figures 3–5f show the results of stacked histograms over 5 days for each patient. The means ± SD of the stacked histograms at ${\theta}_{\mathrm{typ}}$ are summarized in Table 2.

**Table 2:** The mean ± SD values of ΔWET$ {}^{\boldsymbol\theta }$ [mm] from stacked 2d histograms for representative patients

	Beam angles [deg]					
Patients	0	30	60	90	120	150
2	1.2 ± 7.3	0.2 ± 8.9	−2.6 ± 14.5	−3.3 ± 11.3	−0.1 ± 4.5	−0.7 ± 3.3
5	21.4 ± 13.1	4.8 ± 15.0	−1.8 ± 3.8	−3.6 ± 1.5	−3.6 ± 1.7	−7.0 ± 2.3
8	1.3 ± 1.3	1.7 ± 2.0	−4.0 ± 12.4	−1.9 ± 4.6	2.5 ± 2.0	−0.4 ± 2.3
	Beam angles [deg]					
Patients	180	210	240	270	300	330
2	1.2 ± 3.0	−0.6 ± 2.6	−0.6 ± 2.8	0.0 ± 7.0	5.9 ± 14.7	4.1 ± 11.1
5	−10.3 ± 2.4	−6.7 ± 2.3	−11.5 ± 4.7	3.8 ± 3.7	27.3 ± 16.0	11.5 ± 12.6
8	−2.7 ± 2.8	−2.8 ± 3.3	1.2 ± 2.5	0.5 ± 4.2	−0.4 ± 3.7	0.6 ± 1.7

Figure 3 shows the histograms for Patient 2. In the daily 2D polar histograms (Fig. 3a–e), both the mean and variance of daily $\Delta \mathrm{WE}{\mathrm{T}}^{\theta }$ were small around posterior directions from 135° to 225°. The daily $\Delta \mathrm{WE}{\mathrm{T}}^{\theta }$ distributions were widely spread at the anterior directions and varied from day to day around the left (90°) and right (270°) lateral directions. The stacked histogram (f) shows that the $\Delta \mathrm{WE}{\mathrm{T}}^{\theta }$ was distributed near zero around the posterior directions.

The histograms for Patient 5, who had a target on the pancreatic tail, are shown in Fig. 4. The histograms (Fig. 4a–e) show that the daily $\Delta \mathrm{WE}{\mathrm{T}}^{\theta }$ was distributed with less variation around the left lateral and posterior directions on each day, but had greater variation at the other directions, particularly from 300° to 30°. At a beam angle of 90°, the mean values of daily $\Delta \mathrm{WE}{\mathrm{T}}^{\theta }$ were small, showing a maximum deviation of −4.4 mm (fraction 1) and a minimum deviation of −2.8 mm (fraction 3). By contrast, at 180°, the mean values of daily $\Delta \mathrm{WE}{\mathrm{T}}^{\theta }$ showed relatively large deviation, with a maximum of −11.5 mm (fraction 5) and a minimum of −8.4 mm (fraction 3). The mean ± SD values of the stacked histogram were −3.6 ± 1.5 mm at the left lateral beam angle (90°) and −10.3 ± 2.4 mm at 180° (Table 2).

The results for Patient 8, who had received surgical resection of stomach cancer, are shown in Fig. 5. The 2D polar histograms for Patient 8 show that the daily $\Delta \mathrm{WE}{\mathrm{T}}^{\theta }$ was distributed within ~±5 mm for overall beam angles except ~60°, where the GI tract located on the beam path to the CTV (Fig. 5a–e). Similarly, the stacked histograms exhibited a consistent trend, with small deviation across most angles. Indeed, the mean values in the stacked histograms remained within ±5 mm for all ${\theta}_{\mathrm{typ}}$, and the standard deviation was small for ${\theta}_{\mathrm{typ}}$ except 60° (Table 2).

**Fig. 6:**
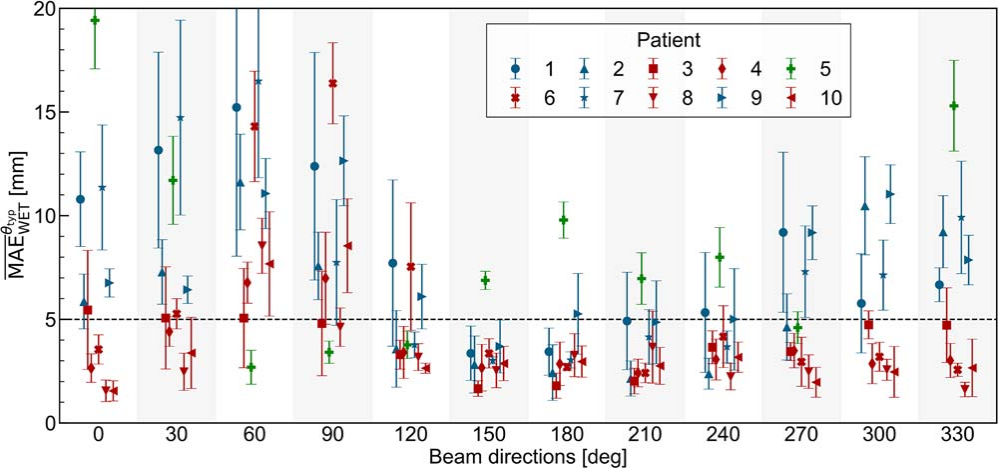
Error bar plot illustrating the mean and SD of MAE over 5 days for all 10 patients, with groups formed based on ${\boldsymbol\theta}_{\mathbf{typ}}$ with ${\overline{\mathbf{MAE}}}_{\mathbf{WET}}^{\boldsymbol\theta_{\mathbf{typ}}}$ < 5 mm.

Angular trends in the interfractional ARVs were quantitatively evaluated using ${\overline{\mathrm{MAE}}}_{\mathrm{WET}}^{\theta_{\mathrm{typ}}}$ and ${\overline{\mathrm{R}}}_{\mathrm{pass}}^{\theta_{\mathrm{typ}}}$. Figure 6 shows the mean and SD of ${\overline{\mathrm{MAE}}}_{\mathrm{WET}}^{\theta_{\mathrm{typ}}}$ over 5 days for each of the 10 patients. Generally, the mean of ${\overline{\mathrm{MAE}}}_{\mathrm{WET}}^{\theta_{\mathrm{typ}}}$ for 10 patients over 5 days was smaller around the posterior directions, with values of 3.3 mm at a beam angle of 150°, 3.6 mm at 210° and 3.8 mm at 180° in order of smaller value. Conversely, larger values were observed around anterior or lateral directions, with values of 9.9 mm at 60°, 7.5 mm at 300° and 7.4 mm at 30°, in order of larger value. We also focused on the individual angular trends of interfractional ARVs, guided by a criterion of ${\overline{\mathrm{MAE}}}_{\mathrm{WET}}^{\theta_{\mathrm{typ}}}$ <5 mm following the previous studies^13,19^. Subsequently, three distinct angular trends of interfractional ARVs were observed from the 10 patients. Patients 1, 2, 7 and 9 (group 1) showed a ${\overline{\mathrm{MAE}}}_{\mathrm{WET}}^{\theta_{\mathrm{typ}}}$ <5 mm around the posterior directions, whereas Patients 3, 4, 6, 8 and 10 (group 2) showed a trend toward smaller ${\overline{\mathrm{MAE}}}_{\mathrm{WET}}^{\theta_{\mathrm{typ}}}$ for most angles except for 60° and 90°. For Patient 5 (group 3), smaller ${\overline{\mathrm{MAE}}}_{\mathrm{WET}}^{\theta_{\mathrm{typ}}}$ was observed in left lateral directions.

**Fig. 7:**
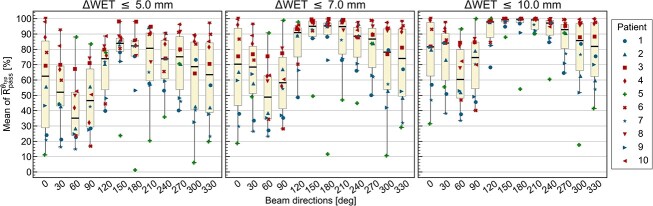
A boxplot representing the mean of ${\overline{\mathbf{R}}}_{\boldsymbol{pass}}^{\boldsymbol\theta_{\mathbf{typ}}}$ for all 10 patients. The results for each patient are plotted using marker of the same shape as in Fig. 6.

Figure 7 shows boxplots of the mean of ${\overline{\mathrm{R}}}_{\mathrm{pass}}^{\theta_{\mathrm{typ}}}$ over 5 days for each threshold. The points represent 10 patients, and the color of each point represents the same classification, as shown in Fig. 6. The mean values of ${\overline{\mathrm{R}}}_{\mathrm{pass}}^{\theta_{\mathrm{typ}}}$ for all patients were clearly higher around the posterior directions except for Patient 5. With a threshold of 5 mm, in descending order of the number of patients achieving ${\overline{\mathrm{R}}}_{\mathrm{pass}}^{\theta_{\mathrm{typ}}}$ ≥80%, 7 out of 10 patients met the criterion at a beam angle of 150°, 6 patients at 180° and 5 patients at 210°. When the threshold was increased from 5 to 7 and 10 mm, the ${\overline{\mathrm{R}}}_{\mathrm{pass}}^{\theta_{\mathrm{typ}}}$ clearly improved around the posterior directions. With a threshold of 7 mm, 9 out of 10 patients met the same criterion at a beam angle of 150°, 8 patients at 180° and 7 patients at 120°, 210°, 240° and 270°. Similarly, with a threshold of 10 mm, all patients met the same criterion at 150° and 210° and nine patients met the same criterion at 120°, 180° and 240°. The trend of the ${\overline{\mathrm{R}}}_{\mathrm{pass}}^{\theta_{\mathrm{typ}}}$ was similar to the ${\overline{\mathrm{MAE}}}_{\mathrm{WET}}^{\theta_{\mathrm{typ}}}$. That is, the patients in group 1 displayed a high ${\overline{\mathrm{R}}}_{\mathrm{pass}}^{\theta_{\mathrm{typ}}}$ around posterior directions, the patients in group 2 exhibited a relatively higher ${\overline{\mathrm{R}}}_{\mathrm{pass}}^{\theta_{\mathrm{typ}}}$ than the other groups at most beam directions except 60 and 90, and Patient 5 showed a high ${\overline{\mathrm{R}}}_{\mathrm{pass}}^{\theta_{\mathrm{typ}}}$ around the left lateral beam directions.

### Impact of interfractional ARVs on dose distribution

All patients were evaluated daily dose distribution between Plan A, using beam angles, in which the target was closest to the body surface, and Plan B, which selected beam angles with small interfractional ARVs. Beam angles of 0° and 90° were selected for Plan A of Patients 3, 5, 6 and 10. For other patients, beam angles of 0° and 270° were selected for Plan A. In the case of Plan B, posterior beam angles of 150° and 210° were chosen for all patients except the Patient 5. They were 60° and 90° for Patient 5 in Plan B.

**Fig. 8:**
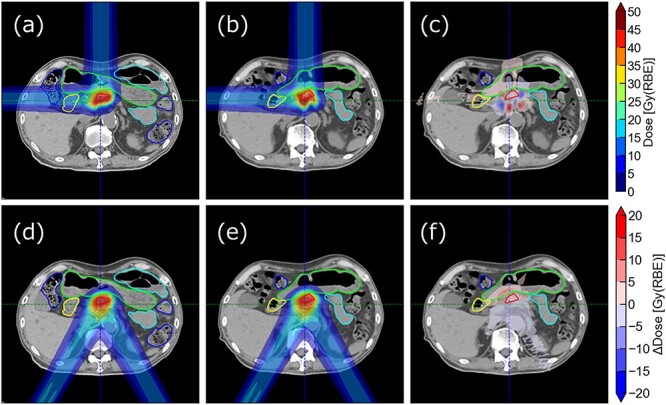
Dose distributions for the Patient 2. Panels (**a**), (**b**) and (**c**) show the dose distributions of the treatment plan, fraction 1 and the distribution of their difference for the Plan A, respectively. Panels (**d**), (**e**) and (**f**) present the corresponding dose distributions for Plan B.

Figure 8 shows the dose distribution of treatment plan, fraction 1, and the dose difference for Patient 2 for each plan. In Plan A, interfractional anatomical changes led to large interfractional ARVs and distorted the dose distribution. Notably, in the 0° direction, a large ΔWET variance of 7.3 mm (Table 2) resulted in distortion in the high dose region. By contrast, Plan B resulted in trivial variations in dose distribution. For Patient 5, Plan A demonstrates a substantial distortion of dose distribution (Supplementary Fig. S1). Particularly, a 0° beam, which passed through the GI tract upstream of the CTV, resulted in a proximal shift in the dose distribution; this shift led to a large dose reduction on the left side of the CTV. In Plan B, which used a 60° beam to avoid the GI tract and achieve smaller interfractional ARVs than the 0° beam, the dose variation was smaller than that in Plan A. For Patient 8, despite not considering ARVs in Plan A, the interfractional ARVs were small at the beam directions selected in Plan A (0° and 270°), as shown in Fig. 5. Therefore, both plans for Patient 8 resulted in a minimal dose distortion compared with the other patients (Supplementary Fig. S2).

Supplementary Table S1 summarizes the DVH parameters. The plans that included beam directions with large interfractional ARVs indicated large reductions in target coverage. As for OAR sparing, no obvious difference was observed between Plans A and B. For example, for Patient 2 in Plan A, the $\Delta{D}_{99}$ of the CTV decreased by a minimum of −4.6 Gy(RBE) and a maximum of −24.5 Gy(RBE). In contrast, Plan B, which selected beam directions with small interfractional ARVs, showed a small reduction of only −2.2 Gy(RBE). In Plan B, the maximum deviation for Stomach+5 mm was ${V}_{38\mathrm{Gy}}$ = 1.9 cc owing to interfractional ARVs because the OAR was located on the distal side of the target, which received a high dose; however, this maximum deviation was comparable with that of Plan A (1.7 cc). In Patient 5, despite using different beam angles from Patient 2, similar trends were observed. Plan B consistently showed less reduction in target coverage than Plan A. Unlike the results seen in Patients 2 and 5, Patient 8 showed only slight differences in DVH parameters between Plans A and B. For Patient 8, both plans satisfied almost constraints for the target, with an average $\Delta{D}_{99}$ of −4.0 Gy (RBE) for Plan A and −2.4 Gy (RBE) for Plan B. Regarding OAR sparing, neither plan deviated from the constraints.

**Fig. 9:**
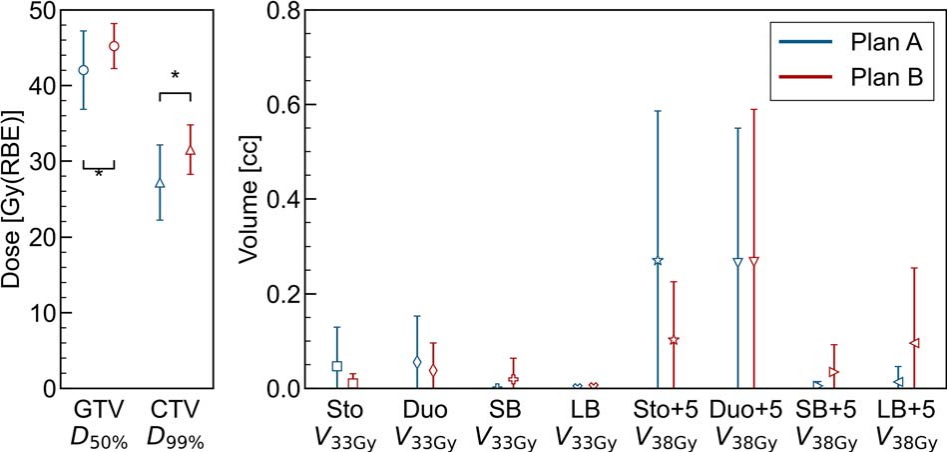
Error bar plot showing the mean and SD of the averaged DVH parameters for all patients. The averaged DVH parameters represent the averages of the 5-day data for each patient. The asterisks (*) indicate statistically significant differences (*P* < 0.05) between Plans A and B, as determined by the paired t-test.

Figure 9 showed that the mean and SD of the averaged DVH parameters for all patients. The averaged DVH parameters represent the averages of the 5-day data for each patient. We performed paired t-test to statistically analyze the differences in the averaged DVH parameters between Plans A and B for all 10 patients. The statistical analysis revealed that the daily mean value of CTV ${D}_{99}$ and GTV ${D}_{50}$ were significantly smaller in Plan B compared with Plan A (*P* < 0.05). These results indicate that selecting beam angles with smaller interfractional ARVs effectively minimizes the decline in target coverage. In terms of DVH parameters for OAR, no significant differences were observed between the plans.

## DISCUSSION

Herein, we visualized patient-specific daily ΔWET in 2D polar histograms and quantitatively evaluated the trend of the interfractional ARVs using ${\overline{\mathrm{MAE}}}_{\mathrm{WET}}^{\theta_{\mathrm{typ}}}$ and ${\overline{\mathrm{R}}}_{\mathrm{pass}}^{\theta_{\mathrm{typ}}}$ on each beam direction. The results revealed that interfractional ARVs were generally small around the posterior directions for patients with pancreatic cancer. This result is consistent with those from previous studies and supports using posterior beam angles for treatment planning to minimize dose distortion caused by interfractional ARVs in proton therapy of pancreatic cancer [6, 7]. In addition, we performed a more detailed patient-specific evaluation and determined the characteristics of individual interfractional ARVs. Patients were visually classified into three groups, each demonstrating a distinct trend (Figs 6 and 7). Patients in group 1 showed small interfractional ARVs primarily around the posterior direction with large interfractional ARVs observed at other directions. The 2D polar histogram of Patient 2 (Fig. 3) in group 1 clearly demonstrated this trend. The patients in group 1 experienced substantial daily variations of gas in the GI tract. The observed angular trend of interfractional ARVs in group 1 would attribute to this anatomical variation. In group 2, the patients showed small interfractional ARVs at most directions, except ~60° and 90°. Compared with patients in group 1, those in group 2 had relatively smaller volumes of gas in the GI tract. Additionally, this group also included patients who had received surgical resection for abdominal region (i.e. Patients 6 and 8). The reduced volume of the GI tract on the beam path due to surgery could contribute to the smaller interfractional ARVs across most beam directions for these patients. In group 3, which only contained Patient 5, showed minimal interfractional ARVs particularly ~90°. This trend could be attributed to the unique situation of Patient 5. That is, the target was located in the pancreatic tail and close to the body surface from the left lateral beam directions. Although investigating a large cohort may reveal additional patterns, three distinct patterns from 10 patients shown in this study suggest a correlation between the angular trends of interfractional ARVs and the anatomical structures.

The ΔWET from the body surface to the isocenter plane was evaluated for our study, whereas range variation studies often calculate the ΔWET from the body surface to the distal end of target volume (e.g. CTV) [9–12, 18]. For small target volumes eligible for SBRT, the ΔWET to the isocenter plane is not greatly different from the ΔWET to the distal end of the CTV. In the cases evaluated in our study, the distance from the isocenter plane to the distal end of the CTV was ~<1 cm. Additionally, because the main factors of the interfractional ARVs could be changes in body surface and gas in the GI tract, the interfractional ARVs mainly occurred on the proximal side of the target volume. Therefore, our ΔWET evaluation could suffice as a practical approach; nevertheless, the change in target volume is also required for considering target coverage, particularly for the larger target volumes.

The impact of interfractional ARVs on daily dose distribution was evaluated from two beam selection strategies: Plan A, using beam angles, in which the target was closest to the body surface, and Plan B, using beam angles with small interfractional ARVs. This evaluation was based on the angular trends observed in interfractional ARV evaluation. For example, in Patients 2 and 5, large ARVs in the 0° direction led to a large coverage reduction in Plan A. Conversely, in Patient 8, small ARVs in the 0° direction maintained target coverage in Plan A comparable with Plan B. This difference can be attributed to whether the beam passes through the GI tract; this is, for Patients 2 and 5, 0° beam passes through the GI tract, whereas for Patient 8, GI tract is not located upstream of the target in 0° direction. These results indicate that the beam directions with less impact on target coverage varied among patients, suggesting that selecting beam angles with minimal interfractional ARVs for each patient will enable robust irradiation. Recent studies have investigated interfractional range variations and their impact on dose distributions with beam angles for pancreatic cancer patients in carbon ion therapy, taking a statistical approach that highlights overall trends across a broad patient base [13]. This methodology offers valuable insights into general patterns. We sought to analyze the patient-specific interfractional ARVs with beam angles for pancreatic patients, which enabled us to identify beam angles with small interfractional ARVs tailored to the individual tendencies of each patient. Selecting those beam angles can take the advantages of gantry with beam angle flexibility and reduce target coverage loss significantly in proton therapy. This finding differentiates our study from previous studies and further emphasizes the importance of evaluating patient-specific ARVs with beam directions. Since interfractional ARVs cannot be calculated during the planning phase, if interfractional ARVs could be predicted from the positional relationship of anatomical structures, it would contribute to the selection of beam angles with robust irradiation for each individual patient.

We focused on criteria of ${\overline{\mathrm{MAE}}}_{\mathrm{WET}}^{\theta_{\mathrm{typ}}}$ <5 mm and ${\overline{\mathrm{R}}}_{\mathrm{pass}}^{\theta_{\mathrm{typ}}}$ >80% for the ΔWET <5 mm threshold, similar to the prior research. Chang *et al.* achieved robust IMPT plans for range variation due to intrafractional motion in thoracic cancer by selecting the beam angle based on the percentage of ΔWET <5 mm in a voxel of CTV being >80% [19]. Yu *et al.* concluded that $\Delta{D}_{95}$ was <1% when the mean of ΔWET was <5 mm in cases of esophageal cancer [20]. Although these previous studies focused on different anatomical sites, our study demonstrates that such range variation criteria can also estimate the impact of interfractional ARVs on dose distribution in pancreatic cancer cases. In Plan B for Patients 2 and 5, and both plans for Patient 8, ${\overline{\mathrm{MAE}}}_{\mathrm{WET}}^{\theta_{\mathrm{typ}}}$ for both beam directions were <5 mm and $\Delta{D}_{99}$ was relatively small. Conversely, Plan A for Patients 2 and 5 included beam directions with ${\overline{\mathrm{MAE}}}_{\mathrm{WET}}^{\theta_{\mathrm{typ}}}$ >5 mm, resulting in a large coverage loss of $\Delta{D}_{99}$ >10 Gy(RBE) (Supplementary Table S1). Regarding ${\overline{\mathrm{R}}}_{\mathrm{pass}}^{\theta_{\mathrm{typ}}}$, similar trends were observed. It is noted that further research is needed to quantitatively determine specific thresholds, however our evaluation shows consistent results with previous studies and suggests that these indicators are plausible for interfractional ARVs in pancreatic cancer cases [19, 20].

In pancreatic cancer, the relative position changes between the gold fiducial marker and vertebrae can be significant, with difference of >10 mm [18]. In our study, there were differences of >10 mm between marker matching and bone matching in 12 out of all 50 fractions. These shifts can lead to substantial range variations caused by the vertebrae when using posterior beam angles. However, it is important to note that, in almost cases, the range variations caused by the vertebrae are relatively smaller compared with those caused by GI tract, as shown in ${\overline{\mathrm{MAE}}}_{\mathrm{WET}}^{\theta_{\mathrm{typ}}}$ and ${\overline{\mathrm{R}}}_{\mathrm{pass}}^{\theta_{\mathrm{typ}}}$ (Figs 6 and 7). This finding is consistent with our dose evaluation, which suggests that posterior beam angles can still be advantageous despite the potential impact of vertebrae. In addition, while our study provides insights for small pancreatic cancer targets (CTV volume: 2.2–20.7 cc), further validation is needed for larger targets, such as the 118–342 cc volumes evaluated by Narita *et al.* [8]. Future research should explore the applicability of our findings to larger target volumes to ensure comprehensive treatment planning strategies.

Despite the promising findings, our study had some limitations. First, we used a dataset from 10 patients only, but a larger dataset may be needed to evaluate trends in patients with whole pancreatic cancer because of the large uncertainty in anatomical changes around the pancreas [7, 8, 18]. We utilized all eligible cases with dCT imaging every fraction at our institution between September 2020 and July 2022. We acknowledge that the limited sample size poses a challenge for generalizing our findings to the entire population of pancreatic cancer patients. Nonetheless, our comprehensive evaluation using two different planning strategies on 50 dCT images for each fraction demonstrates the importance of selecting beam angles with minimal range variations to mitigate coverage loss. This detailed analysis underscores the significance of our findings, even within the constraints of a limited sample size. Second, few limitations were present in the reproducibility of breath holding during CT scans. To address this issue at our hospital, four-dimensional CT (4DCT) scans were taken before the breath-hold CT scans, and it has been confirmed that the marker position error in expiratory phase of 4DCT and breath-hold CT were within ±2 slices (~±4 mm) at most. Therefore, the impact of the reproducibility on the range evaluation in this study is considered to be sufficiently small. Then, there are also slight differences in patient positioning between pCT and dCT images, the dose evaluation in this study involved matching the pCT and dCT images using the same alignment protocol as employed clinically during treatment. Consequently, the setup errors have been sufficiently negligible. Finally, we only evaluated the physical indicators of range variations and dose distribution with constant RBE = 1.1 but not biological effects with the variable RBE. The importance of variable RBE comprising linear energy transfer (LET), cell type, and particle type has been reported in treatment outcome [21, 22]. Irradiation from the posterior directions can deliver the distal end of beam with high LET to the abdominal OARs in pancreatic cancer cases. Biological effects also need to be investigated to reach a sufficient consensus on selecting beam angles for pancreatic cancer. However, even when investigating biological effects under the influence of variable RBE, our findings remain crucial for minimizing variations in physical dose owing to interfractional ARVs.

## CONCLUSION

We quantitatively investigated interfractional ARVs with beam directions based on the $\Delta \mathrm{WE}{\mathrm{T}}^{\theta }$ from body surface to isocenter plane and the impact of ARVs on dose distribution in proton therapy for pancreatic cancer. Our results indicate the patient-specific variation in the angular trends of interfractional ARVs. While posterior beam angles generally exhibit smaller interfractional ARVs, individual variations need us more personalized planning strategy. Selecting beam angles with small interfractional ARVs for each patient enhances the robustness of dose distribution, reducing target coverage loss. These findings emphasize the need for a beam angle selection in proton therapy, accounting for individual anatomical changes to optimize treatment outcomes.

## Supplementary Material

Fig_S1_rrae069

Fig_S2_rrae069

Table_S1_revised_rrae069
