# Supplementary figures and images for: Investigation of interfractional range variation owing to anatomical changes with beam directions based on water equivalent thickness in proton therapy for pancreatic cancer
Source: J Radiat Res. 2024 Oct 8;65(6):813–23. doi: 10.1093/jrr/rrae069 (PMC11629986; doi:10.1093/jrr/rrae069)

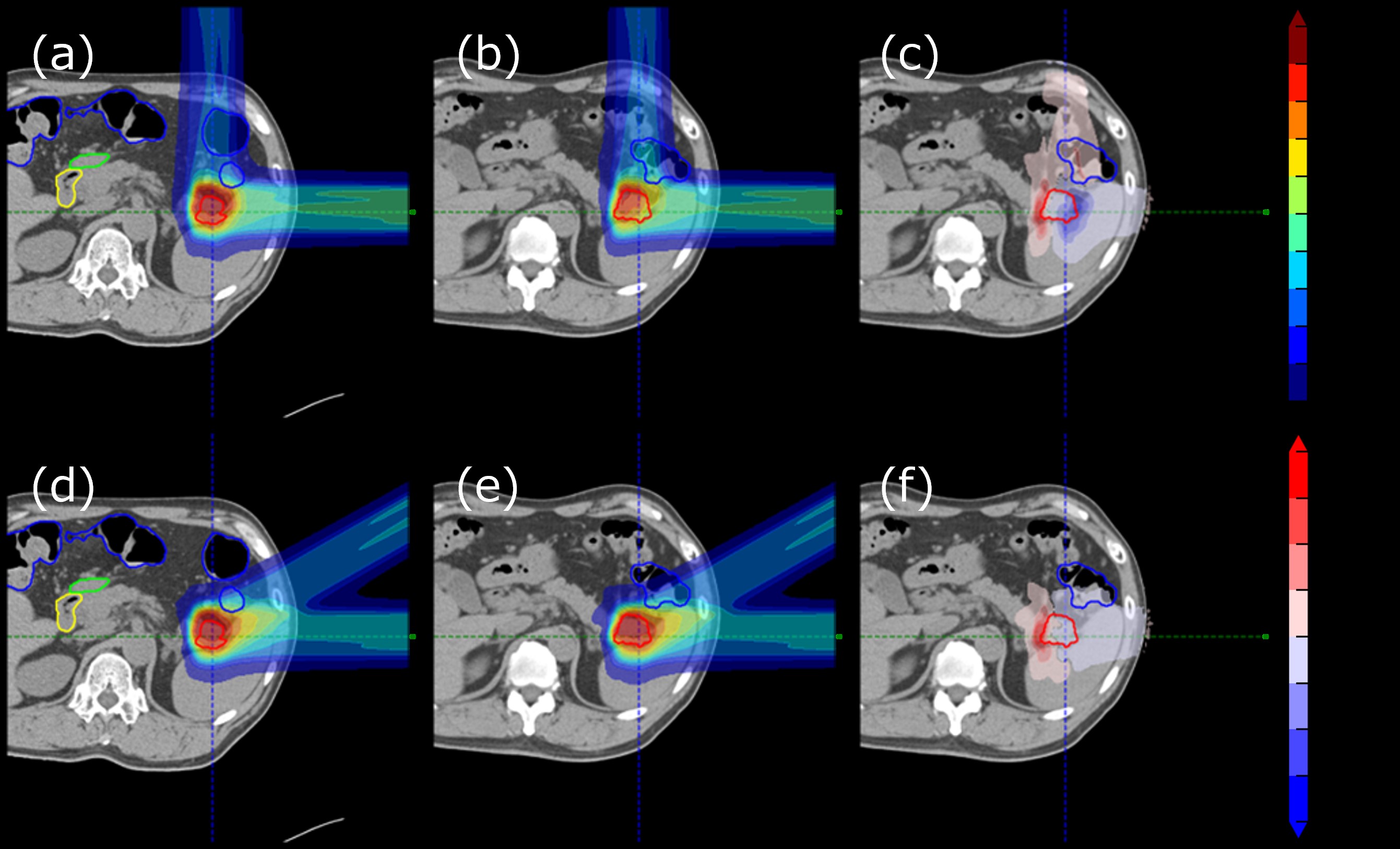

Supplement: Fig_S1_rrae069 [file fig_s1_rrae069.jpeg]

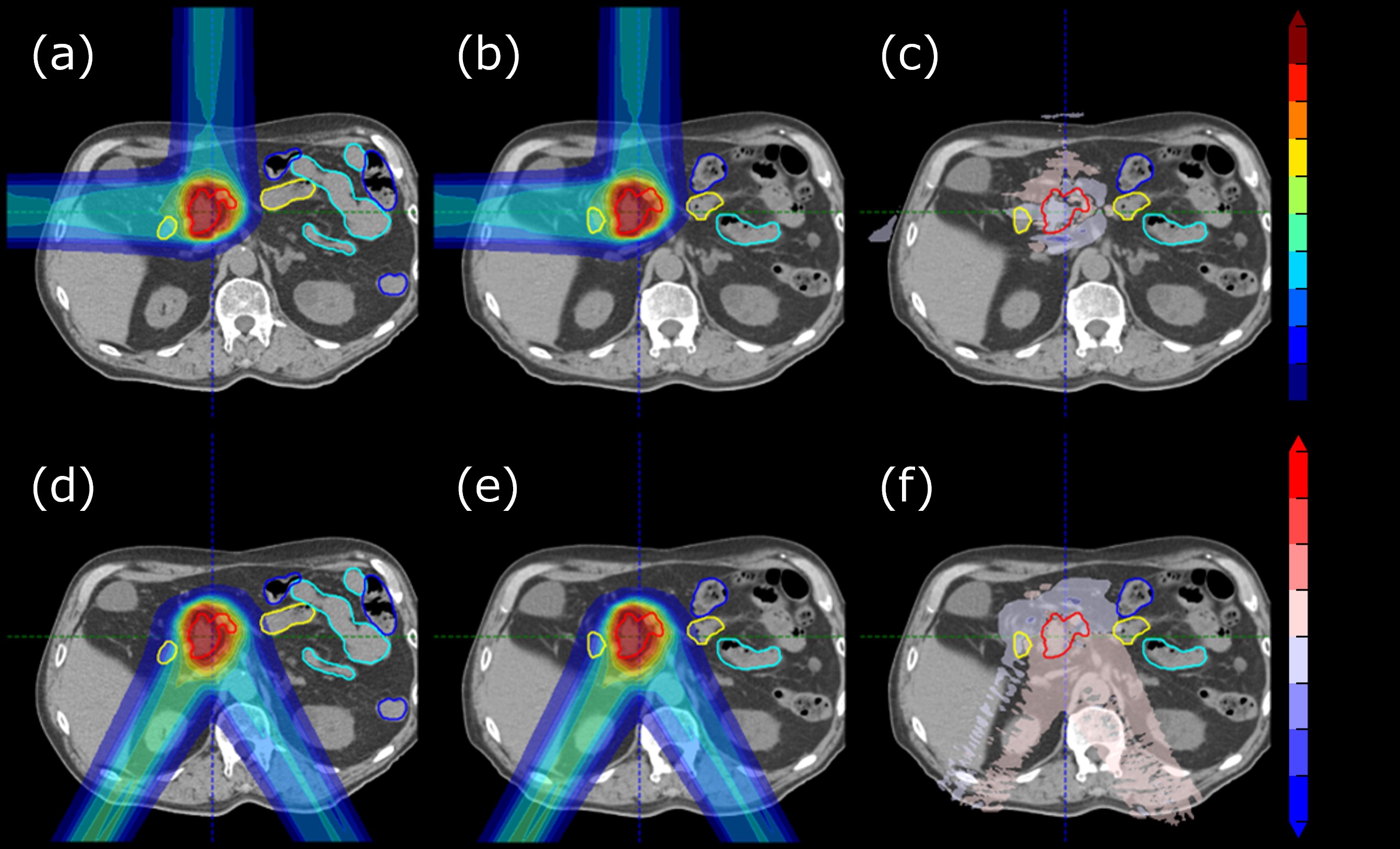

Supplement: Fig_S2_rrae069 [file fig_s2_rrae069.jpeg]
