# Supplementary material for: Investigation of interfractional range variation owing to anatomical changes with beam directions based on water equivalent thickness in proton therapy for pancreatic cancer
Source: J Radiat Res. 2024 Oct 8;65(6):813–23. doi: 10.1093/jrr/rrae069 (PMC11629986; doi:10.1093/jrr/rrae069)
Supplement: Table_S1_revised_rrae069 [file table_s1_revised_rrae069.docx]

|  |  | Patient 1 |  | Patient 2 |  | Patient 3 |  | Patient 4 |  | Patient 5 |  |
| --- | --- | --- | --- | --- | --- | --- | --- | --- | --- | --- | --- |
| Condition | Constraint | A(0$^{\circ}$, 270$^{\circ}$) | B(150$^{\circ}$, 210$^{\circ}$) | A(0$^{\circ}$, 270$^{\circ}$) | B(150$^{\circ}$, 210$^{\circ}$) | A(0$^{\circ}$, 90$^{\circ}$) | B(150$^{\circ}$, 210$^{\circ}$) | A(0$^{\circ}$, 270$^{\circ}$) | B(150$^{\circ}$, 210$^{\circ}$) | A(0$^{\circ}$, 90$^{\circ}$) | B(60$^{\circ}$, 90$^{\circ}$) |
| GTV | ${\Delta D}_{50\%}$ [Gy(RBE)] | −9.1  (−11.9, −7.2) | −4.0  (−7.7, −1.2) | −2.7  (−5.3, −0.5) | −0.7  (−1.2, 0.0) | −0.5  (−0.9, −0.1) | −0.3  (−0.5, −0.2) | −0.4  (−0.5, −0.2) | −0.4  (−0.7, 0.0) | −6.9  (−8.8, −5.3) | −0.9  (−1.3, −0.6) |
| CTV | ${\Delta D}_{99\%}$ [Gy(RBE)] | −8.0  (−11.0, −3.0) | −8.1  (−13.1, −3.0) | −12.5  (−24.5, −4.6) | −0.5  (−2.2, 1.5) | 0.1  (−1.3, 1.7) | −1.2  (−3.0, 0.3) | −5.2  (−7.9, −2.5) | −2.9  (−6.5, −1.3) | −10.9  (−12.2, −9.8) | −2.7  (−6.8, 2.5) |
| Stomach | $V_{33\mathrm{Gy}}$ [cc] | 0.1  (0.0, 0.3) | 0.2  (0.0, 0.8) | 0.3  (0.0, 0.8) | 0.1  (0.0, 0.3) | 0.0  (0.0, 0.0) | 0.0  (0.0, 0.0) | 0.0  (0.0, 0.0) | 0.0  (0.0, 0.0) | 0.0  (0.0, 0.1) | 0.0  (0.0, 0.2) |
| Duodenum |  | 0.0  (0.0, 0.0) | 0.0  (0.0, 0.0) | 0.0  (0.0, 0.0) | 0.0  (0.0, 0.0) | 0.0  (0.0, 0.0) | 0.0  (0.0, 0.0) | 0.2  (0.1, 0.3) | 0.0  (0.0, 0.1) | - | - |
| Small Bowel |  | 0.0  (0.0, 0.0) | 0.0  (0.0, 0.0) | 0.0  (0.0, 0.0) | 0.0  (0.0, 0.0) | 0.0  (0.0, 0.0) | 0.0  (0.0, 0.0) | 0.0  (0.0, 0.0) | 0.0  (0.0, 0.0) | 0.0  (0.0, 0.0) | 0.0  (0.0, 0.0) |
| Large Bowel |  | 0.0  (0.0, 0.0) | 0.0  (0.0, 0.1) | - | - | 0.0  (0.0, 0.0) | 0.0  (0.0, 0.0) | 0.0  (0.0, 0.0) | 0.0  (0.0, 0.1) | 0.0  (0.0, 0.0) | 0.1  (0, 0.6) |
| Stomach+5mm | $V_{38\mathrm{Gy}}$ [cc] | - | - | 0.7  (0.0, 1.7) | 0.9  (0.0, 1.9) | 0.4  (0.0, 0.6) | 0.3  (0.0, 0.7) | 0.0  (0.0, 0.0) | 0.0  (0.0, 0.0) | 0.3  (0.1, 0.6) | 0.7  (0.3, 1.4) |
| Duodenum+5mm |  | - | - | 0.0  (0.0, 0.0) | 0.0  (0.0, 0.0) | 0.0  (0.0, 0.0) | 0.0  (0.0, 0.0) | 0.9  (0.3, 1.2) | 0.3  (0.0, 0.7) | - | - |
| Small Bowel+5mm |  | - | - | 0.0  (0.0, 0.0) | 0.0  (0.0, 0.0) | 0.0  (0.0, 0.1) | 0.2  (0.0, 0.5) | 0.0  (0.0, 0.0) | 0.0  (0.0, 0.2) | 0.0  (0.0, 0.0) | 0.0  (0.0, 0.0) |
| Large Bowel+5mm |  | - | - | - | - | 0.0  (0.0, 0.0) | 0.0  (0.0, 0.0) | 0.0  (0.0, 0.0) | 0.2  (0.0, 0.7) | 0.1  (0.0, 0.4) | 0.4  (0.0, 1.6) |
|  |  | Patient 6 |  | Patient 7 |  | Patient 8 |  | Patient 9 |  | Patient 10 |  |
| Condition | Constraint | A(0$^{\circ}$, 90$^{\circ}$) | B(150$^{\circ}$, 210$^{\circ}$) | A(0$^{\circ}$, 270$^{\circ}$) | B(150$^{\circ}$, 210$^{\circ}$) | A(0$^{\circ}$, 270$^{\circ}$) | B(150$^{\circ}$, 210$^{\circ}$) | A(0$^{\circ}$, 270$^{\circ}$) | B(150$^{\circ}$, 210$^{\circ}$) | A(0$^{\circ}$, 90$^{\circ}$) | B(150$^{\circ}$, 210$^{\circ}$) |
| GTV | ${\Delta D}_{50\%}$ [Gy(RBE)] | −15.8  (−23.7, −1.1) | −1.7  (−2.6, −1.2) | −3.7  (−7.5, −1.1) | −0.1  (−0.4, 0.1) | −0.2  (−0.4, 0.0) | 0.2  (−0.2, 0.3) | −0.2  (−1.8, 2.1) | 0.5  (0.0, 1.2) | −5.4  (−6.7, −3.1) | −1.9  (−3.5, −0.3) |
| CTV | ${\Delta D}_{99\%}$ [Gy(RBE)] | −20.2  (−27.3, −13.1) | −7.0  (−11.0, −3.8) | −8.0  (−15.5, −4.4) | −5.6  (−12.6, −3.2) | −4.0  (−6.2, −1.2) | −2.4  (−4.9, −0.7) | −3.3  (−7.6, 2.1) | −2.9  (−9.3, −0.3) | −13.8  (−16.5, −9.1) | −12.2  (−15.1, −8.4) |
| Stomach | $V_{33\mathrm{Gy}}$ [cc] | 0.0  (0.0, 0.1) | 0.0  (0.0, 0.1) | 0.0  (0.0, 0.0) | 0.0  (0.0, 0.0) | 0.0  (0.0, 0.0) | 0.0  (0.0, 0.0) | 0.2  (0.0, 0.6) | 0.0  (0.0, 0.0) | 0.0  (0.0, 0.0) | 0.0  (0.0, 0.0) |
| Duodenum |  | 0.0  (0.0, 0.0) | 0.0  (0.0, 0.0) | 0.0  (0.0, 0.1) | 0.0  (0.0, 0.0) | 0.0  (0.0, 0.0) | 0.0  (0.0, 0.0) | 0.1  (0.0, 0.3) | 0.1  (0.0, 0.1) | 0.0  (0.0, 0.0) | 0.0  (0.0, 0.0) |
| Small Bowel |  | - | - | 0.0  (0.0, 0.0) | 0.0  (0.0, 0.0) | 0.0  (0.0, 0.0) | 0.0  (0.0, 0.0) | 0.0  (0.0, 0.0) | 0.0  (0.0, 0.0) | 0.0  (0.0, 0.0) | 0.0  (0.0, 0.0) |
| Large Bowel |  | - | - | - | - | 0.0  (0.0, 0.0) | 0.0  (0.0, 0.0) | 0.0  (0.0, 0.0) | 0.0  (0.0, 0.0) | 0.0  (0.0, 0.0) | 0.0  (0.0, 0.0) |
| Stomach+5mm | $V_{38\mathrm{Gy}}$ [cc] | 0.4  (0.0, 0.9) | 0.3  (0.1, 0.7) | 0.0  (0.0, 0.2) | 0.2  (0.0, 0.4) | 0.0  (0.0, 0.0) | 0.0  (0.0, 0.0) | 0.4  (0.0, 0.9) | 0.1  (0.0, 0.3) | 0.0  (0.0, 0.1) | 0.0  (0.0, 0.0) |
| Duodenum+5mm |  | 0.3  (0.0, 0.8) | 0.2  (0.0, 0.4) | 0.5  (0.2, 0.7) | 0.1  (0.0, 0.2) | 0.1  (0.0, 0.4) | 0.0  (0.0, 0.0) | 0.4  (0.3, 0.7) | 0.3  (0.1, 0.4) | 0.0  (0.0, 0.0) | 0.0  (0.0, 0.0) |
| Small Bowel+5mm |  | - | - | 0.0  (0.0, 0.0) | 0.0  (0.0, 0.0) | 0.0  (0.0, 0.0) | 0.0  (0.0, 0.0) | 0.0  (0.0, 0.0) | 0.0  (0.0, 0.0) | 0.0  (0.0, 0.1) | 0.0  (0.0, 0.0) |
| Large Bowel+5mm |  | - | - | - | - | 0.0  (0.0, 0.0) | 0.0  (0.0, 0.0) | 0.0  (0.0, 0.0) | 0.0  (0.0, 0.0) | 0.0  (0.0, 0.0) | 0.0  (0.0, 0.0) |
